# Supplementary material for: Single-molecule, full-length transcript isoform sequencing reveals disease-associated RNA isoforms in cardiomyocytes
Source: Nat Commun. 2021 Jul 9;12:4203. doi: 10.1038/s41467-021-24484-z (PMC8270901; doi:10.1038/s41467-021-24484-z)
Supplement: Supplementary file 8 — Reporting Summary [file 41467_2021_24484_MOESM8_ESM.pdf]

nature portfolio

Corresponding author(s): Lars Steinmetz
Wu Wei

Last updated by author(s): Jun 9, 2021

Reporting Summary

Nature Portfolio wishes to improve the reproducibility of the work that we publish. This form provides structure for consistency and transparency in reporting. For further information on Nature Portfolio policies, see our Editorial Policies and the Editorial Policy Checklist.

Statistics

For all statistical analyses, confirm that the following items are present in the figure legend, table legend, main text, or Methods section.

n/a	Confirmed	
☐	☒	The exact sample size (*n*) for each experimental group/condition, given as a discrete number and unit of measurement
☐	☒	A statement on whether measurements were taken from distinct samples or whether the same sample was measured repeatedly
☐	☒	The statistical test(s) used AND whether they are one- or two-sided<br>*Only common tests should be described solely by name; describe more complex techniques in the Methods section.*
☒	☐	A description of all covariates tested
☐	☒	A description of any assumptions or corrections, such as tests of normality and adjustment for multiple comparisons
☐	☒	A full description of the statistical parameters including central tendency (e.g. means) or other basic estimates (e.g. regression coefficient) AND variation (e.g. standard deviation) or associated estimates of uncertainty (e.g. confidence intervals)
☐	☒	For null hypothesis testing, the test statistic (e.g. *F*, *t*, *r*) with confidence intervals, effect sizes, degrees of freedom and *P* value noted<br>*Give P values as exact values whenever suitable.*
☒	☐	For Bayesian analysis, information on the choice of priors and Markov chain Monte Carlo settings
☒	☐	For hierarchical and complex designs, identification of the appropriate level for tests and full reporting of outcomes
☒	☐	Estimates of effect sizes (e.g. Cohen's *d*, Pearson's *r*), indicating how they were calculated

Our web collection on statistics for biologists contains articles on many of the points above.

Software and code

Policy information about availability of computer code

Data collection	ONT's MinKNOW (1.4.2) and Albacore (version 2.1.3)
Data analysis	Minimap2 (version version: 2.9-r751-dirty), Star (v2.5.lb), featureCounts (1.6.0), Kallisto (v0.46.2), DESeq2 (1.26.0), custom scripts based on bash, R (3.5.3), python (https://github.com/czhu/FulQuant)

For manuscripts utilizing custom algorithms or software that are central to the research but not yet described in published literature, software must be made available to editors and reviewers. We strongly encourage code deposition in a community repository (e.g. GitHub). See the Nature Portfolio guidelines for submitting code & software for further information.

Data

Policy information about availability of data

All manuscripts must include a data availability statement. This statement should provide the following information, where applicable:

- Accession codes, unique identifiers, or web links for publicly available datasets
- A description of any restrictions on data availability
- For clinical datasets or third party data, please ensure that the statement adheres to our policy

The long-read data reported in this study have been deposited in ArrayExpress under accession number E-MTAB-7334. The short-read data have been deposited in Sequence Read Archive under accession number PRJNA579336. Furthermore, our genome-wide full-length isoform dataset can be viewed with our custom genome browser at http://steinmetzlab.embl.de/iBrowser/.

Field-specific reporting

Please select the one below that is the best fit for your research. If you are not sure, read the appropriate sections before making your selection.

- [x] Life sciences
- [ ] Behavioural & social sciences
- [ ] Ecological, evolutionary & environmental sciences

For a reference copy of the document with all sections, see nature.com/documents/nr-reporting-summary-flat.pdf

Life sciences study design

All studies must disclose on these points even when the disclosure is negative.

Sample size	iPSC differentiation into cardiomyocytes was done in duplicates. Two mutants were compared against wildtype. Duplicates were chosen based on available material. It is typical for performing differential analysis with RNA-seq
Data exclusions	Reads were filtered based on mean quality score (cutoff 6). For differential analysis only genes and transcripts with more than 10 read counts in total from all samples were considered. Before cutoffs were previously not established
Replication	iPSC-CM and associated RNA-seq were performed in duplicates. Biological replicates had similar expression profiles as assessed by scatter plots and correlation coefficients.
Randomization	Samples were not randomized in this study. All samples share the same genetic background except for the mutation of interest. Hence we didn't include any covariantes when performing the comparison
Blinding	We performed unbiased genome-wide analysis on the expression profiles. For iPSC-CM blinding was not possible because we had to introduce the mutations.

Reporting for specific materials, systems and methods

We require information from authors about some types of materials, experimental systems and methods used in many studies. Here, indicate whether each material, system or method listed is relevant to your study. If you are not sure if a list item applies to your research, read the appropriate section before selecting a response.

Materials & experimental systems

n/a	Involved in the study	
x		Antibodies
	x	Eukaryotic cell lines
x		Palaeontology and archaeology
x		Animals and other organisms
x		Human research participants
x		Clinical data
x		Dual use research of concern

Methods

n/a	Involved in the study	
x		ChIP-seq
x		Flow cytometry
x		MRI-based neuroimaging

Eukaryotic cell lines

Policy information about cell lines

Cell line source(s)	SCVI-15S1 from Stanford Cardiovascular Institute Biobank. Human iPSC- RBM20 P633L, Human iPSC- RBM20 R634Q and Human iPSC- RBM20 WT
Authentication	R634Q and P633L mutations were verified by whole genome sequencing
Mycoplasma contamination	not tested
Commonly misidentified lines (See ICLAC register)	no commonly misidentified lines were used in this study
