# Supplementary material for: Single-molecule, full-length transcript isoform sequencing reveals disease-associated RNA isoforms in cardiomyocytes
Source: Nat Commun. 2021 Jul 9;12:4203. doi: 10.1038/s41467-021-24484-z (PMC8270901; doi:10.1038/s41467-021-24484-z)
Supplement: Supplementary file 9 — Description of Additional Supplementary Files [file 41467_2021_24484_MOESM9_ESM.pdf]

**Title:** Supplementary Data 1.
**Description:** ONT sequencing runs with yield for all samples.

**Title:** Supplementary Data 2.
**Description:** All identified transcripts isoforms with exon coordinates in BED format.

**Title:** Supplementary Data 3.
**Description:** Quantification (read counts) of all transcript isoforms in all samples.

**Title:** Supplementary Data 4.
**Description:** Significantly co-associated exon pairs.

**Title:** Supplementary Data 5.
**Description:** List of differentially expressed transcript isoforms between wildtype and mutants.

**Title:** Supplementary Data 6.
**Description:** List of oligos for validating novel splice events with fragment analysis and validation status.
